# Regulation of flagellar motility and biosynthesis in enterohemorrhagic *Escherichia coli* O157:H7

**DOI:** 10.1080/19490976.2022.2110822

**Published:** 2022-08-16

**Authors:** Hongmin Sun, Min Wang, Yutao Liu, Pan Wu, Ting Yao, Wen Yang, Qian Yang, Jun Yan, Bin Yang

**Affiliations:** TEDA Institute of Biological Sciences and Biotechnology, Nankai University, TEDA, Tianjin, China

**Keywords:** EHEC O157:H7, flagellum-mediated motility, flagellar biosynthesis, flagellar gene expression, regulation, molecular mechanism, small regulatory RNAs, environmental factors

## Abstract

Enterohemorrhagic *Escherichia coli* (EHEC) O157:H7 is a human pathogen that causes a variety of diseases, such as hemorrhagic colitis and lethal hemolytic uremic syndrome. Flagellum-dependent motility plays diverse roles in the pathogenesis of EHEC O157:H7, including its migration to an optimal host site, adherence and colonization, survival at the infection site, and post-infection dispersal. However, it is very expensive for cellular economy in terms of the number of genes and the energy required for flagellar biosynthesis and functioning. Furthermore, the flagellar filament bears strong antigenic properties that induce a strong host immune response. Consequently, the flagellar gene expression and biosynthesis are highly regulated to occur at the appropriate time and place by different regulatory influences. The present review focuses on the regulatory mechanisms of EHEC O157:H7 motility and flagellar biosynthesis, especially in terms of flagellar gene regulation by environmental factors, regulatory proteins, and small regulatory RNAs.

## Introduction

Enterohemorrhagic *Escherichia coli* (EHEC) O157:H7 is a foodborne pathogen that causes severe diseases, including diarrhea, hemorrhagic colitis (HC), and hemolytic uremic syndrome (HUC).^1^,^2,3^ It has three major virulence factors, including Shiga toxins (Stxs), products of the pathogenicity island called the locus of enterocyte effacement (LEE), and products of the large virulence plasmid pO157.^4^ Stxs are bacteriophage-encoded AB5 family toxins that cause damage to a variety of cell types and have often been associated with HC and the lethal hemolytic uremic syndrome (HUS) in humans.^5^ LEE consists of five polycistronic operons (LEE1 to LEE5) encoding a type III secretion system and associated effectors and enables the bacteria to intimately adhere to host epithelial cells.^4^ pO157, a highly conserved non-conjugated plasmid with a size ranging from 92 to 104 kb,^6^ is known to harbor several genes involved in the pathogenesis of EHEC O157:H7 infections, including *etpC-etpO, ehxA, toxB, katP, espP*, and *stcE* .^7^

**Figure 1.**
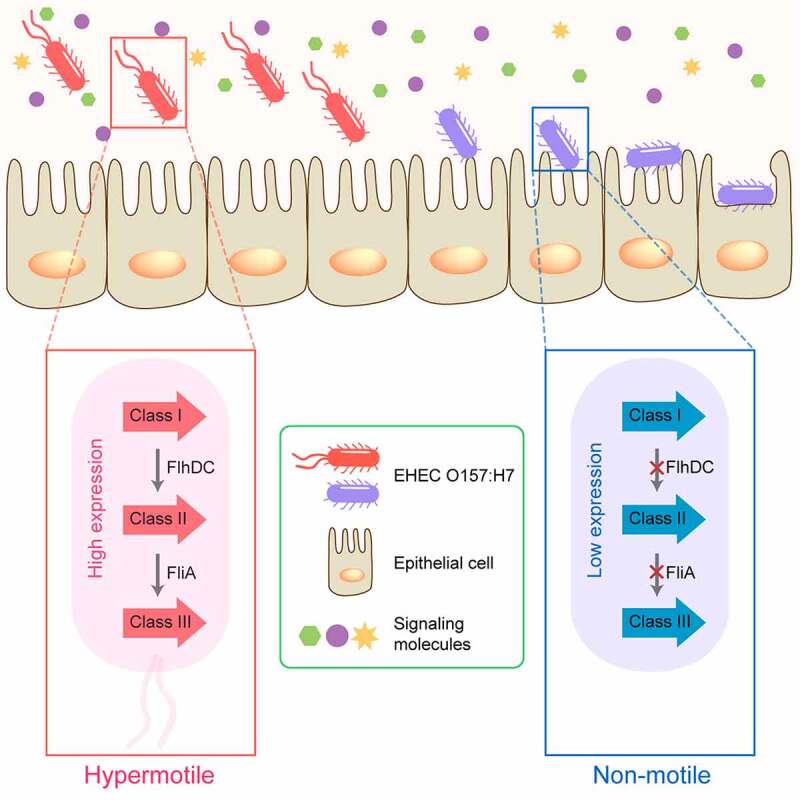
Flagellar regulation during enterohemorrhagic *Escherichia coli* (EHEC) O157:H7 interaction with the host cells. Upon entering the host intestine, flagellum-mediated motility enables EHEC O157:H7 to reach and adhere to optimal colonization sites in the host. After successful infection, EHEC O157:H7 inhibits flagellar biosynthesis to minimize unnecessary energy expenditure and host immune response.

The bacterial flagellum is a macromolecular machine that consists of a basal body (rotary motor), a hook (universal joint), and a filament (propeller).^8^ Flagellar-mediated motility confers an important advantage for bacteria in moving toward favorable conditions or in avoiding detrimental environments and allows bacteria to pursue nutrients and to reach and maintain their preferred niches for survival.^9^ In addition to having locomotive properties, flagellum-mediated motility plays diverse roles in the pathogenesis and progression of EHEC O157:H7 infection. Upon entering the host intestine, EHEC O157:H7 relies on flagellum-mediated motility to reach and adhere to optimal colonization sites in the host.^10^ Subsequently, EHEC O157:H7 inhibits flagellar biosynthesis to save energy and minimize host immunity (Figure 1).^10^

**Figure 2.**
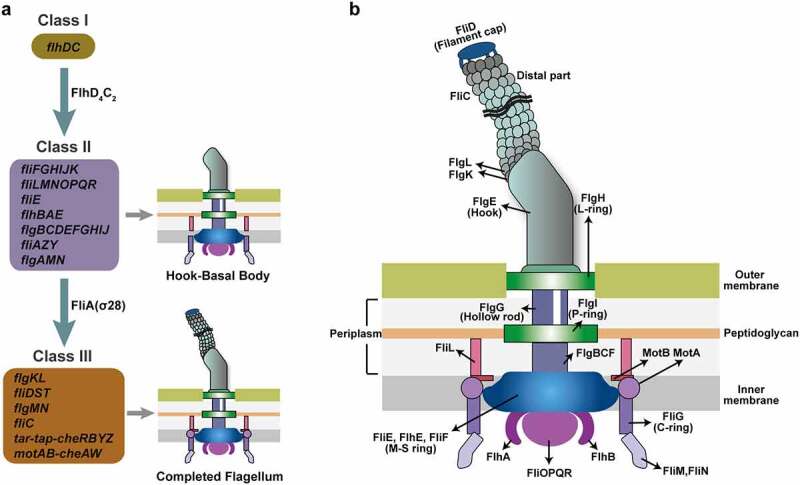
Flagellar genes and structure of EHEC O157:H7. (a) The genetically defined hierarchy of flagellar genes. More than 50 flagellar genes are arranged in a regulatory cascade of three classes (Class I, II, and III). The Class I gene *flhDC* encodes the master regulator of Class II genes. Class II genes are responsible for the synthesis of hook-basal body and two competing regulatory proteins (FlgM and FliA) of Class III genes, and Class III genes are involved in the synthesis of complete flagellum and chemotaxis system. (b) Schematic showing the major components of the flagella (basal body, hook, and filament) and the location of the flagellar gene products.

Flagellar biosynthesis is an energy-intensive process requiring 2% of the cell’s biosynthetic resources and flagellar rotation consumes 0.1% of the cell’s energy.^11^ More than 50 genes constituting at least 14 operons are involved in the biosynthesis and assembly of a flagellum.^12,13^ These flagellar genes are divided into three hierarchical classes (Class I, II, and III) based on their timing and mode of expression (Figure 2a).^14^ The Class I gene *flhDC* encodes the master flagellar regulator, with FlhD and FlhC forming a heterotetramer (FlhD_4_C_2_).^14^ Class II genes, including *fliFGHIJK, fliLMNOPQR, fliE, flhBAE, flgBCDEFGHIJ, fliAZY*, and *flgAMN*, encode structural and assembly proteins required for the biosynthesis of the hook-basal body and a pair of flagellar regulatory proteins, FliA and FlgM (Figure 2a, b).^13,15^ Class III genes, including *flgKL, fliDST, flgMN, fliC, tar-tap-cheRBYZ*, and *motAB-cheAW*, encode cell-distal structural components of the flagellum and flagellar function (rotation and chemotaxis) (Figure 2a, b).^14,16^ The expression of flagellar genes is tightly regulated, with FlhDC, FliA, and FlgM playing major regulatory roles in the flagellar transcription network. Flagellar biosynthesis is also regulated by various environmental signals, regulatory proteins, and small RNAs (sRNA) by modulating *flhDC* and/or *fliA* transcription or translation. In the present review, we provide a broad overview of the regulatory mechanisms of flagellar biosynthesis and motility in EHEC O157:H7, especially focusing on the regulatory function of environmental factors, transcriptional regulatory proteins, post-transcriptional and post-translation regulatory proteins, and small regulatory RNAs on flagellar gene expression at both transcriptional and translational levels.

## Three-tiered flagellar regulatory cascade

**Figure 3.**
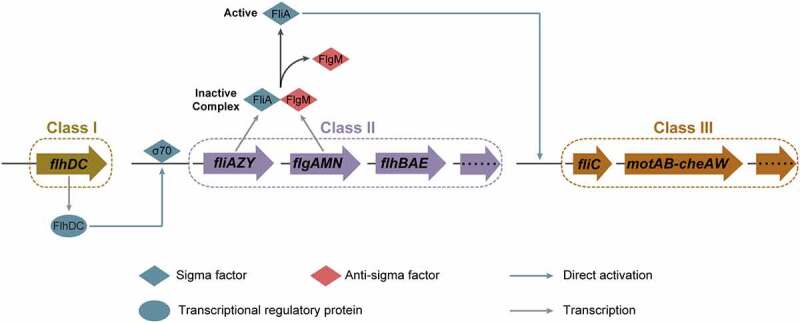
Three-tiered flagellar regulatory cascade in EHEC O157:H7. In the three-tiered flagellar regulatory cascade, FlhDC promotes σ^70^-dependent transcription of Class II genes, FliA acts as an alternative sigma factor (σ^28^) specifically required for the transcription of Class III genes, and FlgM acts as an anti-sigma factor that binds to FliA and inhibits its activity.

In *E. coli*, including EHEC O157:H7, the expression of flagellar genes is a tightly regulated and highly energetic three-tier process (Figure 3).^12^ The Class I gene *flhDC* encodes proteins FlhD and FlhC, that assemble into the heterohexamer (FlhD_4_C_2_).^14^ The FlhD_4_C_2_ proteins complex binds to the DNA upstream of Class II genes, recruits RNA polymerase, and promotes σ^70^-dependent transcription (Figure 3).^17^ FliA is an alternate sigma factor (σ^28^), encoded by a Class II gene, specifically required for transcription initiation of Class III genes.^18^ FlgM acts as an anti-sigma factor that binds to FliA directly, preventing interaction with RNA polymerase and repressing FliA-dependent transcription until after hook-basal body are formed.^19^ Upon assembly of the basal body and secretion apparatus, FlgM is exported out of the cell, freeing FliA and allowing initiation of FliA-dependent transcription from Class III promoters.^20^ This three-tiered flagellar regulatory cascade helps bacteria, including EHEC O157:H7, to conserve biosynthetic resources and ensure the efficiency of flagellar assembly.

## Environmental factors affecting EHEC O157:H7 motility and flagellar biosynthesis

**Figure 4.**
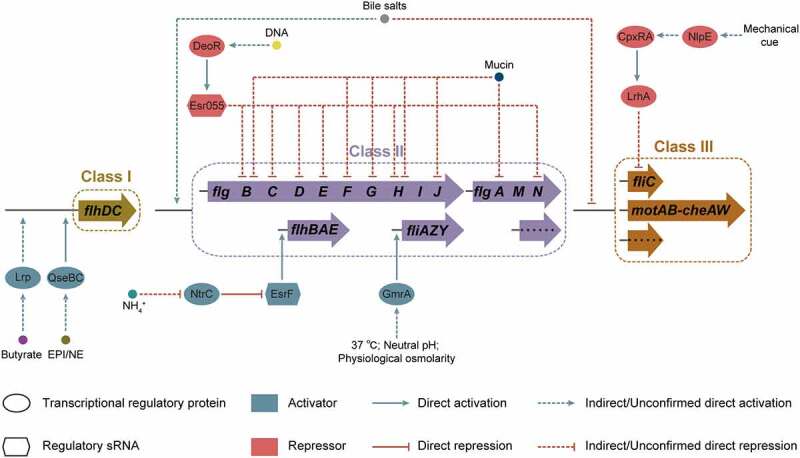
Regulation of flagellar gene expression by environmental factors in EHEC O157:H7. Environmental signals, including butyrate, mucin, bile salts, epinephrine/norepinephrine (EPI/NE), mechanical cue, temperature, pH, osmolarity, and DNA and ammonium concentrations, affect EHEC O157:H7 flagellar motility and biosynthesis.

EHEC O157:H7 can survive and persist in varied environments, such as soil, water, and food, as well as in host gastrointestinal tract,^21^ for which it requires the ability to adapt to variations or extreme changes in conditions commonly encountered in nature. Flagellum-mediated motility allows EHEC O157:H7 to move away from stressful environments and to reach environments with optimal nutrients for survival.^9^ The motility and flagellar biosynthesis of EHEC O157:H7 are affected by diverse environmental factors that include short-chain fatty acids (SCFAs), mucin, bile salts, epinephrine/norepinephrine, indole, and mechanical cues (Figure 4 and Table 1).

**Table 1.** Regulation of EHEC O157:H7 flagellar motility and biosynthesis by environmental factors, regulatory proteins and regulatory sRNAs.

Types	Flagellar motility and biosynthesis-related functions	References
**Environmental factors**		
Short-chain fatty acids	Butyrate activates the *flhDC* gene through Lrp, while butyrate, acetate, and propionate activate the downstream flagellar genes independently of *flhDC* activation.	^22,23^
Mucin	Mucin negatively regulates EHEC O157:H7 motility by repressing the expression of the *flg* genes encoding the components of the flagella basal body at the transcriptional level.	^24^
Bile salts	Bile salts upregulate the expression of the genes encoding components of the basal body-hook structure and downregulate the expression of the genes encoding the elements of the flagellar filament and motor stator.	^25^
Epinephrine/norepinephrine	Epinephrine/norepinephrine positively regulates flagellar-mediated motility via QseBC in EHEC O157:H7. Addition of 50 µM epinephrine or norepinephrine to motility agar plates significantly increased EHEC O157:H7 motility.	^26,27^
Indole	High concentrations of indole (e.g., 500 μM) decrease EHEC O157:H7 motility; however, the underlying mechanism remains unclear.	^27^
Mechanical cue	EHEC O157:H7 perceives attachment to hydrophobic glass beads and host cells as a mechanical cue that leads to downregulated expression of the *fliC*. This mechanical cue-mediated flagellar gene regulation is mediated by NlpE, CpxRA, and LrhA.	^28^
**Transcriptional regulatory proteins**		
Activators		
GmrA	GmrA directly binds to the promoter of *fliA* and thereby upregulates flagellar genes controlled by FliA.	^29^
PchE	PchE upregulates genes in different flagellar operons, including regulatory, structural, chemotaxis, and motor genes, and stimulates EHEC O157:H7 motility.	^30^
Z5898	OI-172-encoded Z5898 promotes *fliC* expression at the transcriptional level by increasing the *fliC* promoter activity.	^31^
Hha	Hha upregulates EHEC O157:H7 motility and flagellar biosynthesis directly by binding to the *flhDC* promoter as well as indirectly by controlling *qseBC* transcription.	^32,33^
Repressors		
GrlA	GrlA negatively regulates flagellar gene expression by reducing the transcription of the *flhDC* operon in EHEC O157:H7.	^34^
FmrA	OI-1-encoded FmrA regulates motility and flagellar biosynthesis by interacting with the FlhDC complex and preventing it from binding to promoters of Class II flagellar genes.	^35^
IHF	IHF indirectly represses *flhDC* via a putative regulator that is not encoded by LEE in EHEC O157:H7. In addition, the IHF-mediated repression of *flhDC* is unique to EPEC and EHEC.	^36^
SdiA	SdiA negatively regulates the motility and *fliC* expression via Hha in EHEC O157:H7.	^37,38^
CadA	CadA negatively regulates the expression of 16 flagellar genes, including *fliA, flgABCDEFGH* and *fliEFGJLMN*, and this regulation may be dependent on FliA.	^39^
AdhE	AdhE represses the expression of all three flagellar gene classes, including the regulatory genes (*flhC, flhD*, and *fliA*), structural genes, and genes encoding chemotaxis machinery and energy transduction systems.	^40^
RcsB	RcsB represses EHEC O157:H7 motility and *flhDC* transcription, and this RcsB-dependent repression is positively regulated by GrlA at the post-transcriptional level.	^41^
Two-component regulatory system and other systems		
QseBC	QseC responds to epinephrine/norepinephrine to autophosphorylate and transfer of the phosphate to QseB. The phosphorylated QseB upregulates EHEC O157:H7 flagellar gene expression by directly interacting with the *flhDC* regulatory region at distal high-affinity and proximal low-affinity binding sites.	^42–44^
Tol-Pal system	The Tol-Pal system, consisting of TolQ, TolR, TolA, TolB, and Pal proteins, promotes EHEC O157:H7 motility and *fliC* expression through an unknown mechanism.	^45,46^
NlpE-CpxRA-LrhA	NlpE senses a mechanical cue generated by surface attachment and signals it to the two-component regulatory system CpxRA. The activated CpxR directly binds to the *lrhA* promoter and activates its expression. LrhA, in turn, indirectly represses the expression of *fliC*.	^28^
Epigenetic regulatory proteins		
MraW	MraW directly binds to flagellar gene sequences, including *fliJ, fliR, fliK*, and *fhiA*, and increases methylation levels, which in turn promote gene expression.	^47^
**Post-transcriptional and post-translational regulatory proteins**		
CsrA	CsrA positively regulates the expression of 53 flagella-related genes, comprise genes required for the synthesis and assembly of flagella as well as transcriptional regulators of flagellar gene expression such as *flhD, flhC, flgM*, and *fliA*. The *csrA::kan* mutant is non-motile on semi-solid agar plates.	^48^
ClpXP	ClpXP protease downregulates EHEC O157:H7 motility and flagellar gene expression through two pathways, namely post-translational degradation of the FlhDC master regulator and transcriptional control of the *flhDC* operon through the LEE-encoded GrlR-GrlA regulatory system.	^49^
Hfq	Hfq indirectly activates EHEC O157:H7 motility and flagellar gene expression though the post-transcriptional regulation of *qseBC* and *grlA*.	^50–52^
**Small regulatory RNAs**		
EsrF	EsrF promotes the expression of several flagellar genes, including *flhB, flhD, flhC, fliA*, and *fliC*, and thus enhances EHEC O157:H7 motility. Among these EsrF-regulated flagellar genes, *flhB* was identified as the direct target of EsrF. EsrF binds to the 5′ untranslated region (UTR) of the *flhB* mRNA, which may release the Rho-dependent termination of the *flhB* 5′ UTR, resulting in increased *flhB* expression.	^53^
Esr055	Esr055 represses the expression of six flagellar genes, including *flgB, flgC, flgD, flgE, flgH*, and *flgN*, although the underlying mechanism remains unclear. The expression of Esr055 is directly activated by DeoR in response to high concentrations of DNA.	^54^
Esr41	Esr41 activates the expression of Class III flagellar genes by indirectly inducing the transcription of *fliA* via an unknown transcriptional regulator and thereby enhances EHEC O157:H7 motility.	^55,56^
MavR	MavR promotes flagellar gene expression by post-transcriptionally influencing the expression of FlhD in EHEC O157:H7.	^57^

### Short-chain fatty acids

Short-chain fatty acids (SCFAs) are fermentation end-products of dietary fiber metabolism by gastrointestinal microorganisms.^58^ They are mainly composed of acetic, propionic, and butyric acids, with acetic acid being the most abundant.^59^ The concentration and composition of SCFAs in the gastrointestinal tract are uneven, being lower in the small intestine (20–40 mM) and higher in the large intestine (70–200 mM).^22^ The expression of flagellar genes, including *flhC* and *flhD* (the master regulatory genes), motility genes (*motAB*) and chemotaxis gene (*cheA*), and bacterial motility in EHEC O157:H7 were significantly upregulated when exposed to SCFA mixture representative of the small intestine but were downregulated upon exposure to SCFA mixtures representative of the large intestine.^22^ In addition, exposure to 20 mM butyrate, a concentration similar to that of the large intestine SCFA, was reported to enhance the expression of flagellar genes in EHEC O157:H7.^23^ Butyrate, but not acetate and propionate, activated the *flhDC* regulatory genes through the leucine-responsive regulatory protein Lrp.^23^ However, butyrate, acetate, and propionate also activated downstream flagellar genes independently of *flhDC* activation. Therefore, precise regulation of motility and flagellar biosynthesis in response to SCFAs provides EHEC O157:H7 with a profound advantage in transiting through the host gastrointestinal tract, permitting the pathogen to avoid detrimental locations and to find more favorable niches.^23^

### Mucin

The gastrointestinal tract is lined by a continuously secreted mucus layer formed mainly by high-molecular mass (200–2000 kDa) oligomeric mucin glycoproteins.^24^ Mucins act as receptors for bacterial adhesin proteins and provide protection against the adherence of infectious pathogens by steric hindrance or through specific bacterial binding domains.^60,61^ A whole genome-scale transcriptome analysis revealed that 732 candidate genes were differentially expressed in EHEC O157:H7 in the presence of 0.5% porcine stomach mucin.^24^ Of particular interest, eight genes associated with flagellar biosynthesis (including *flgA, flgB, flgF, flgG, flgH, flgJ, fliP*, and *yaiU*) were all downregulated in the presence of mucin. Furthermore, qRT-PCR analyses showed that the expression of six genes in the *flg* operon (i.e., *flgABFGH* and *flgJ*) was downregulated two- to five-folds in the presence of mucin, which further verified the transcriptome analysis results. Consistently, the bacterial motility was also significantly reduced when EHEC O157:H7 was grown on 0.3% tryptone agar plates containing mucin. Therefore, mucin may act as an intestinal environmental signal that negatively regulates EHEC O157:H7 motility through the transcriptional repression of the *flg* gene that encodes the components of the flagellar basal body.^24^

### Bile salts

Bile salts are the main component of human liver bile, with their concentration ranging from 0.2% to 2% in the small intestine.^62^ EHEC O157:H7 encounters acute acid stress and broken bile during its passage through the gastrointestinal tract.^63^ A transcriptome analysis revealed that bile salts induce an unusual effect on the transcript levels of flagellar genes.^25^ The transcript levels of the regulatory genes/operons (*flhDC, flgMN*, and *fliAZY*) controlling flagellar biosynthesis remained relatively unchanged under bile salt treatment compared to that in untreated controls. However, flagellar genes encoding the components of the basal body-hook structure, including *flgA, fliLMNOPQR, fliE, fliFGHIJK, flgBCDEFGHIJ*, and *flhBAE*, had two- to four-folds increased transcription levels in the bile-salt treated cells. In contrast to the basal body-related genes, other flagellar genes, including *fliC, fliDST, flgKL, tar-tap-cheZYBR*, and *motAB-cheAW*, which encode the elements of the flagellar filament, motor stator, and chemotaxis system had approximately twofold reduction in their transcription levels. A significant increase in EHEC O157:H7 motility was observed when 0.8% or 0.1% bile salts were incorporated into the soft agar medium.^25^ However, incorporating bile salts into the soft agar destabilized it, making it less robust. Therefore, the change in motility may be attributed to the differences in the viscosity of the soft agar with and without the addition of bile salts.^25^ Nevertheless, the mechanism by which EHEC O157:H7 senses bile salts to regulate flagellar gene expression remains unclear, and the effect of bile salts on EHEC O157:H7 motility needs further experimental verification.

### Epinephrine/norepinephrine

Quorum sensing is a cell-to-cell signaling system based on the production of hormone-like compounds known as autoinducers (AI),^64^ which allow bacteria to detect their own population as well as the population of other bacterial species present in the same environment.^64^ The host-derived stress hormones epinephrine (EPI) and norepinephrine (NE) are autoinducer analogs that are recognized by the quorum sensing system and are involved in the regulation of flagellar-mediated motility in EHEC O157:H7.^26,27^ The two-fluorophore chemotaxis assay showed a concentration-dependent migration of EHEC O157:H7 toward EPI and NE.^27^ Furthermore, addition of 50 µM EPI or NE to motility agar plates significantly increased EHEC O157:H7 motility, compared to that in the untreated control.^27^ The regulation of EHEC O157:H7 motility by EPI/NE is mediated by the two-component regulatory system QseBC,^26^ which will be discussed later in the review.

### Indole

Indole, produced by both commensal and pathogenic strains of *E. coli*, is an important signaling molecule in host‒microbe interactions in the gastrointestinal tract.^65^ Because commensal *E. coli* produce as much as 600 μM indole in suspension cultures,^66^ and indole has been detected in human feces at comparable concentrations (~250–1100 μM),^67^ it is likely that EHEC O157:H7 are continually exposed to high concentrations of indole in the human intestinal tract. An agarose plug chemotaxis assay showed that EHEC O157:H7 is repelled by indole.^27^ In addition, a high concentration of indole (500 μM) decreased EHEC O157:H7 motility by 2.8-fold.^27^ However, the mechanisms by which EHEC O157:H7 senses indole as an environmental signal to regulate bacterial motility are still largely unknown.

### Mechanical cue

Motility and biofilm development are mutually exclusive events, and the phase of transition from motility to sessile is determined by a sensory transduction mechanism termed surface sensing, which typically involves bacterial flagella.^68^ As a mechanosensor, flagella play a critical role in surface sensing and the initial stages of surface adhesion.^69^ Once attached to a surface, bacteria respond by producing cyclic diguanylate monophosphate (c-diGMP), a second messenger signaling molecule that drives the transition between the free-living and biofilm lifestyle.^70^ EHEC O157:H7 perceives attachment to hydrophobic glass beads and host cells as a mechanical cue that leads to downregulated expression of the flagellin gene (*fliC*).^28^ This mechanical cue-mediated flagellar gene regulation is mediated by NlpE, CpxRA, and LrhA, which will be discussed later in the review.

## Transcriptional regulatory proteins affecting EHEC O157:H7 motility and flagellar biosynthesis

**Figure 5.**
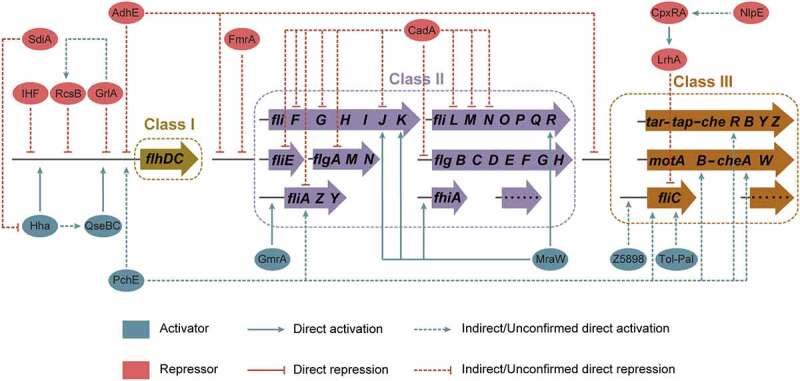
Regulation of flagellar gene expression by transcriptional regulatory proteins in EHEC O157:H7. Transcriptional regulatory proteins, including GmrA, PchE, Z5898, Hha, QseBC, Tol‒Pal system, GrlA, FmrA, IHF, SdiA, CadA, AdhE, RcsB, NlpE‒CpxRA‒LrhA and MraW, affect EHEC O157:H7 flagellar motility and biosynthesis.

EHEC O157:H7 encounters various conditions when colonizing the host intestinal tract or surviving in the external environment.^71^ To ensure its successful survival and colonization, flagellar genes are subjected to strict regulation that allows their expression only under optimal environmental conditions while also avoiding intense metabolic cost or alerting the host immune system.^10^ The coordinated expression of flagellar genes is mainly controlled by transcriptional regulatory proteins, which precisely activate or repress particular genes depending on environmental factors and the stage of infection.^72^ These transcriptional regulatory proteins include GmrA, PchE, Z5898, QseBC, Hha, Tol-Pal system, GrlA, FmrA, IHF, SdiA, CadA, AdhE, RcsB, NlpE-CpxRA-LrhA, and MraW (Figure 5 and Table 1).

### GmrA

Genomic island OI-29 in EHEC O157:H7 is a 2643-bp island that contains the *z0638, z0639* (named *gmrA*), and *z0640* genes, which encode hypothetical proteins of unknown function.^73^ The *gmrA* gene encodes a transcriptional activator required for motility and flagellar biosynthesis in EHEC O157:H7.^29^ A Δ*gmrA* mutant was found to have significantly reduced flagellar biosynthesis and motility on semi-solid LB agar as well as suppressed transcriptional and translational expression of flagellar genes in comparison to the wild-type and complemented strains of EHEC O157:H7. Conversely, the motility and transcriptional expression of flagellar genes were not affected by the deletion of *z0638* and *z0640*. Electrophoretic mobility shift assays (EMSAs), competition experiments, and chromatin immunoprecipitation quantitative PCR (ChIP-qPCR) analysis revealed that GmrA directly binds to the promoter of *fliA*, an RNA polymerase sigma factor, and thereby regulates flagellar genes controlled by FliA. The expression of *gmrA* is maximal in conditions similar to those in the intestinal tract (37°C, neutral pH, and physiological osmolarity) and in the presence of host epithelial cells, indicative of the motility-promoting role of this gene in infection.^29^ A bioinformatics analysis of 231 available *E. coli* genome sequences showed that *gmrA* is highly conserved and widely distributed in various *E. coli* lineages.^29^ In addition, almost all strains with *gmrA* are important pathogens in human or animals, indicating that these hosts might have driven the acquisition of *gmrA* during the evolution of those pathogenic strains. Finally, GmrA was found to be a widespread regulator of bacterial motility and flagellar synthesis in different pathotypes of *E. coli* (including EHEC, enteropathogenic *E. coli*, neonatal meningitis-associated *E. coli*, and avian pathogenic *E. coli*).

### PchE

Five EHEC O157:H7 homologs of the EPEC *perC* gene (*pchA* to *pchE*) have been studied extensively, and a clear role of *pchA, pchB*, and *pchC* in the control of LEE through regulation of *ler* has been demonstrated.^74^ However, *pchE* had little effect on the LEE operons,^74^ and its role in regulating bacterial motility and flagellar gene expression has been recently discovered.^30^ The transcription of *flhC* and *flhD*, which directly or indirectly control the expression of the flagellar operons, were increased >2-fold by *pchE* overexpression in EHEC O157:H7.^30^ In addition, genes in different flagellar operons, including chemotaxis, motor, structural, and regulatory genes, were upregulated by *pchE* overexpression. The expression levels of the *fliA* and *fliC* genes, encoding the RNA polymerase σ^28^ and the flagellar subunit flagellin, respectively, increased by 12- to 24-folds. The expression levels of the motor and chemotaxis genes tested (*motB, cheA*, and *cheR*) were also markedly enhanced by approximately five- to nine-folds. In accordance, the plasmid overexpression of *pchE* increased the diameter of the EHEC O157:H7 motility zone by nearly 50%. However, although *pchE* stimulates flagellar motility, it reduces the adhesion of EHEC O157:H7 to HEP-2 cells, which likely facilitates the movement of EHEC O157:H7 to the intestinal surface in an early stage of infection.^30^

### Z5898

OI-172 is a 44 434-bp genomic island that contains 27 open reading frames (ORFs) encoding several presumed RNA helicases, including a DEAH box RNA helicase (Z5898).^31,73^ A comparative proteomic analysis of wild-type EHEC O157:H7 and Δ*z5898* mutant showed that flagellin was significantly downregulated in the Δ*z5898* mutant.^31^ A motility assay showed that Δ*z5898* mutant migrated slower than the wild-type EHEC O157:H7 and transmission electron microscopy found that approximately 90% Δ*z5898* mutant have no surface flagella. Z5898 promotes *fliC* expression at the transcription level by increasing the *fliC* promoter activity, as indicated by a qRT-PCR analysis and a promoter activity assay that measures green fluorescent protein production. In contrast, the *fliA* transcripts and its promoter activity were not affected by the deletion of *z5898*. Therefore, Z5898 regulates the flagella-based motility by exerting its effect on *fliC* but not its upstream regulator gene, *fliA*. However, the precise mechanisms by which Z5898 activates *fliC* expression are still unclear. Considering Z5898 is an RNA helicase, it is possible that Z5898 is involved in processing *fliC* transcript to increase its expression, which needs further investigation.^31^

### QseBC

The two-component regulatory system sensor protein QseC senses EPI/NE and causes autophosphorylation.^42^ This phosphate is subsequently transferred to the cellular response regulator QseB, and the signals are transmitted to the genome for the differential regulation of signal-sensitive genes. An isogenic *qseC* mutation in EHEC O157:H7 resulted in reduced flagellin expression and motility compared with the wild-type and complemented strains.^43^ The *qseC* mutant showed decreased transcription of *flhD, fliA, motA*, and *fliC*, suggesting that *qseBC* is involved in the transcriptional regulation of flagellar genes. QseBC activates the transcription of *flhDC*, which is the master regulator of the flagella and motility genes, and, in the absence of *flhD*, QseBC is unable to activate the transcription of *fliA*. Further, EMSAs, competition experiments, and DNaseI footprinting analyses showed that the phosphorylated QseB positively regulates EHEC O157:H7 flagellar gene expression by directly interacting with the *flhDC* regulatory region at the distal high-affinity site and the proximal low-affinity binding sites.^44^

### Hha

Hha is a nucleus-associated protein that controls gene expression through DNA structure and by regulating its topology according to osmotic pressure and temperature.^75^ The swimming motility and expression of the flagellar gene *fliC* were significantly reduced in an isogenic Δ*hha* mutant than in the wild-type EHEC O157:H7.^32^ Hha regulates flagellar gene expression by inducing FlhDC, as demonstrated by reduced *flhD* transcription in the Δ*hha* mutant. EMSA results showed that the purified Hha directly binds to the promoter of *flhDC*. The contribution and hierarchy of Hha and QseBC in controlling EHEC O157:H7 motility were also compared. The motility of the Δ*hha* Δ*qseC* double mutant was highly and significantly compromised when compared to the motility phenotypes expressed by the Δ*hha* or Δ*qseC* single mutants.^33^ Complementation with *qseBC*-carrying plasmid increased the motility of the Δ*hha* Δ*qseC* double mutant by a smaller magnitude than complementation of this mutant with the *hha*-carrying plasmid.^33^ These results indicated that Hha exerts greater control than QseBC in regulating the motility of EHEC O157:H7. In addition, Hha is hierarchically superior in the transcriptional regulation of motility than QseBC because the transcription of *qseC* is significantly reduced in the Δ*hha* mutant than that in the wild-type and complemented strains of EHEC O157:H7. Therefore, Hha can regulate EHEC O157:H7 motility and flagellar biosynthesis directly as well as indirectly by controlling the transcription of *qseBC*.^33^

### Tol‒Pal system

The Tol‒Pal system is a protein complex, which was originally characterized by *E. coli* K-12,^76^ and is involved in outer membrane maintenance and uptake of colicin and filamentous phage DNA.^77^ The Tol-Pal system consists of TolQ, TolR, TolA, TolB, and Pal proteins.^78^ In EHEC O157:H7, the inactivation of *tolA*, which encodes the periplasm spanning protein, resulted in complete loss of motility and concomitant transcriptional repression of the major flagellar subunit encoding gene, *fliC*.^45^ Complementation of *tolA* on a low-copy plasmid restored motility and *fliC* transcription to that of wild-type. Similar to the Δ*tolA* mutant, the Δ*tolB* mutant in EHEC O157:H7 also exhibited reduced motility on semi-solid agar compared with the wild-type parent, and the introduction of a heterologous *tolB* expression plasmid increased its motility up to the level of the wild-type parent.^46^ Furthermore, EHEC O157:H7 Δ*tolB* mutant produced defective flagella as determined by microscopy using a 100× objective lens. Consistent with this phenomenon, the western blotting results showed that the flagellin protein, encoded by *fliC*, was undetectable in Δ*tolB* mutant, indicating that the deletion of *tolB* in EHEC O157:H7 decreases FliC levels, which in turn leads to the reduction of flagellar production and motility.^46^ Similarly, the Δ*pal* mutant strain was also less motile and produced fewer flagella than the wild-type EHEC O157:H7 strain.^46^ Thus, these results suggest that the activity of the Tol‒Pal system contributes to EHEC O157:H7 motility and flagellar biosynthesis, although the underlying mechanism is still unclear.

### GrlA

GrlA and GrlR, which are encoded within the LEE, are positive and negative regulators, respectively, of LEE gene.^4,79^ Notably, GrlR is an anti-GrlA factor that inhibits its function by interacting with GrlA.^80^ The GrlR‒GrlA regulatory system not only regulates the expression of LEE genes but also EHEC O157:H7 motility and flagellar biosynthesis.^34^ Both FliC protein levels and bacterial motility were significantly reduced in the Δ*grlR* mutant compared with that in EHEC O157:H7 wild-type strain.^34^ In accordance, the overexpression of *grlA* from a multicopy plasmid strongly represses FliC expression and EHEC O157:H7 motility, indicating that GrlA acts as a negative regulator of flagellar gene expression. Mechanically, GrlA negatively regulates flagellar gene expression by reducing the transcription of the *flhDC* operon in EHEC O157:H7. The constitutively expressed FlhD/FlhC complex inhibits efficient adhesion of EHEC O157:H7 to HeLa cells; therefore, the GrlA-dependent repression of the flagellar regulon may be important for efficient cell adhesion of EHEC O157:H7 to host cells.^34^

### FmrA

Genomic island OI-1 is a putative fimbria-encoding genomic island that contains six ORFs from *z0020* to *z0025*.^35,73^ The deletion of *z0021*, also known as *fmrA* (Fimbrial-Operon-encoded motility regulator A), leads to higher swimming motility and flagellar biosynthesis in EHEC O157:H7 than in the wild-type strain, while the overexpression of *fmrA* inhibits motility.^35^ In contrast, the deletion of other ORFs within OI-1 had no effect on EHEC O157:H7 motility, as the migration distances of the Δ*z0020* and Δ*z0022*‒*z0025* mutants in motility agar plates were comparable to that of wild-type EHEC O157:H7. Transcriptional reporter assays showed that FmrA exerted its regulatory effects prior to Class II and Class III flagellar gene transcription but downstream of the transcription and translation of *flhDC*. Furthermore, the motility defects caused by FmrA can be suppressed by increasing *flhDC* expression, thus implying a genetic link between FmrA and the FlhDC regulatory complexes. It is, therefore, conceivable that FmrA could regulate flagellar biosynthesis by interacting with the FlhDC complex and preventing it from binding to the promoters of Class II flagellar genes.^35^

### Integration host factor (IHF)

IHF is a DNA-binding protein that binds to specific consensus sites and bends the DNA to form nucleoprotein complexes.^81^ IHF is a heterodimer composed of IHF-α and IHF-β subunits that are encoded by the *ihfA* and *ihfB* genes, respectively.^81^ IHF is involved in various cellular processes, including site-specific recombination, DNA replication, transposition, packaging, and gene regulation.^81^ In EHEC O157:H7 and EPEC O127:H6, the *ihfA::kan* mutant was highly motile, and the motility of the strains expressing IHF, either genomic or in trans from a plasmid, was attenuated.^36^ Electron micrographs of the wild-type cells and the *ihfA::kan* mutant grown in Dulbecco’s modified Eagle medium (DMEM) showed that the wild-type cells either completely lack flagella or rarely possess (approximately 5% of bacteria) a single flagellum. In contrast, the *ihfA::kan* mutant produced numerous peritrichous flagella. The IHF-mediated repression of flagellar expression involves the silencing of *flhDC*, which encodes a positive regulator of the flagellar regulon. IHF indirectly represses *flhDC* via a putative regulator that is not encoded by LEE.^36^ In addition, IHF-mediated repression of *flhDC* is unique to EPEC and EHEC and is not found in all *E. coli* strains, such as *E. coli* K-12.^36^

### SdiA

In most quorum-sensing systems, LuxR is required as a transcriptional factor that recognizes and binds to specific QS signaling molecules called autoinducers.^82^ EHEC O157:H7 contains a LuxR homologue called SdiA, which has a helical‒angular‒helical DNA-binding motif.^37^ The overexpression of SdiA from a high-copy-number plasmid significantly reduces the motility of EHEC O157:H7.^37^ A qRT-PCR analysis revealed that the *fliC* expression in the Δ*sdiA* mutant was significantly increased relative to the wild-type EHEC O157:H7.^38^ In contrast, *fliC* expression in the Δ*hha* mutant was reduced compared with that in the wild-type strain, verifying Hha as a positive regulator of bacterial motility. In addition, the ability of the Δ*sdiA* Δ*hha* double mutant to express *fliC* gene showed no significant difference from that of the Δ*hha* single mutant. In other words, *sdiA* deletion had no effect on EHEC O157 *fliC* gene expression in a Δ*hha* mutant background. Therefore, the regulatory role of SdiA for *fliC* gene expression in EHEC O157:H7 is mediated by Hha.^38^

### CadA

The *cadA* gene encodes a lysine decarboxylase that plays a role in EHEC O157:H7 motility and flagellar biosynthesis.^39^ A gene array analysis revealed that 1464 genes (132 upregulated and 1332 downregulated) were differentially regulated by more than twofolds (with 99% confidence) in the Δ*cadA* mutant versus the wild-type strain.^39^ Interestingly, among these most upregulated genes, 16 genes, including *fliA, flgABCDEFGH*, and *fliEFGJLMN*, are involved in flagellar biosynthesis and motility. The qRT-PCR results confirmed the elevated expression of flagellar basal body and hook-encoding genes *flgB* and *flgE*, in concordance with the microarray results. In accordance, an analysis of multiple electron microscopic fields revealed that the Δ*cadA* mutant strain possesses more flagellar filaments than the wild-type strain grown under the same conditions. Interestingly, the motilities of both wild-type EHEC O157:H7 and Δ*cadA* mutant strains were similar on motility agar plates.^39^ The regulatory mechanisms of CadA on EHEC O157:H7 flagellar biosynthesis remain unclear. Considering that most upregulated flagellar genes in Δ*cadA* mutant are under the control of FliA, and the expression of *fliA* was also significantly increased in the Δ*cadA* mutant, the regulation of flagellar genes by CadA may be dependent on FliA.

### AdhE

AdhE is a metabolic enzyme that has the dual enzymatic functions of converting acetyl-CoA to acetaldehyde and acetaldehyde to ethanol.^83^ An RNA-sequence analysis revealed that the deletion of *adhE* in EHEC O157:H7 significantly upregulates all three flagellar gene classes, including the regulatory genes (*flhC, flhD*, and *fliA*), structural genes (such as *fliF, flgE*, and *fliC*), and genes encoding chemotaxis machinery (such as *cheA* and *cheY*) and energy transduction systems (*motA* and *motB*).^40^ In accordance with these results, the SDS-PAGE, tandem mass spectrometry (MS), and immunoblotting analyses showed that the Δ*adhE* mutant exhibited increased expression of FliC, the major structural subunit of the flagellar filament, and FliA, the flagellar-specific sigma factor required for *fliC* transcription. The immunofluorescence microscopy of FliC revealed that the Δ*adhE* mutant with increased levels of FliC exhibited multiple intact flagellar filaments on its surface. Instead, the Δ*adhE* mutant was unable to migrate through semi-solid agar, indicating that the assembled flagella did not rotate. The authors speculated that the assembled flagella are nonfunctional either because a key flagellar component is misassembled and/or some flagellar component is inappropriately modified.^40^

### RcsB

The Rcs phosphorelay system consists of RcsC, RcsB, and RcsA, which encode a sensor kinase, a response regulator, and an auxiliary regulatory protein, respectively.^84^ The Rcs phosphorelay system first identified by its role in the transcriptional regulation of the capsular polysaccharide synthesis genes in *E. coli*.^85^ In addition to capsule synthesis, the Rcs system plays an important role in the regulation of motility and flagellar biosynthesis in EHEC O157:H7.^41^ Compared with that in the wild-type strain, the deletion of *rcsB* enhanced the lateral growth of EHEC O157:H7 on motility plates by 30–40%.^41^ Negative regulation of motility by RcsB has been shown to result from direct transcriptional repression of the global regulator of flagellar biosynthesis and motility genes *flhDC* in *E. coli* K-12.^86^ Consistent with this, the expression of the *flhD* promoter, as measured by *β*-galactosidase activity, was significantly increased in EHEC O157:H7 Δ*rcsB* mutant.^41^ Furthermore, the RcsB-dependent repression of *flhDC* and motility is positively regulated by GrlA at the post-transcriptional level.^41^

### NlpE‒CpxRA‒LrhA

EHEC O157:H7 can adhere to a large variety of surfaces, including glass, metals, many different polymers, as well as to other bacteria and eukaryotic cells.^87^ When EHEC O157:H7 binds to surfaces, the outer-membrane lipoprotein NlpE senses a mechanical cue generated by surface attachment and signals to the sensor kinase CpxA at the inner membrane.^28^ CpxA undergoes autophosphorylation, after which the phosphate group is transferred to the cytoplasmic response regulator CpxR. The phosphorylated CpxR binds directly to the *lrhA* promoter and consequently activates the expression of *lrhA*. LrhA, in turn, indirectly represses the expression of the *fliC* gene. Considering that the LrhA protein bound specifically to the *flhD* promoter in *E. coli* K-12,^88^ and *flhC* expression was increased by the overexpression of LrhA in EHEC O157:H7,^28^ the downregulation of flagellin expression in EHEC O157:H7 in response to adherence to hydrophobic glass beads and host cells might be attributed to the LrhA-induced increase in the levels of FlhDC.^28^

### MraW

MraW is a 16S rRNA methyltransferase that plays an important role in gene regulation through DNA methylation.^89^ It was recently found to contribute to the motility and flagellar production of EHEC O157:H7.^47^ The motility of Δ*mraW* mutant was significantly decreased in correlation with a decrease in the production or secretion of FliC, as determined by western blotting using anti-H7 antisera.^47^ Consistent with decreased motility and flagellin secretion in the Δ*mraW* mutant, the majority (95%) of the Δ*mraW* mutant have no flagella, with the remaining 5% of the cells having one to two flagella. In contrast, most of EHEC O157:H7 wild-type strains possess one to three surface flagella. Whole-genome bisulfite sequencing showed that two flagellar genes (*fliJ* and *fliR*) and two flagellar gene promoters (*fliK* and *fhiA*) had lower levels of methylation in the Δ*mraW* mutant compared to the wild-type strain. Furthermore, MraW has been demonstrated to directly bind to these four flagellar gene sequences and increase methylation levels, which in turn promote gene expression.^47^

## Post-transcriptional and post-translational regulatory proteins affecting EHEC O157:H7 motility and flagellar biosynthesis

**Figure 6.**
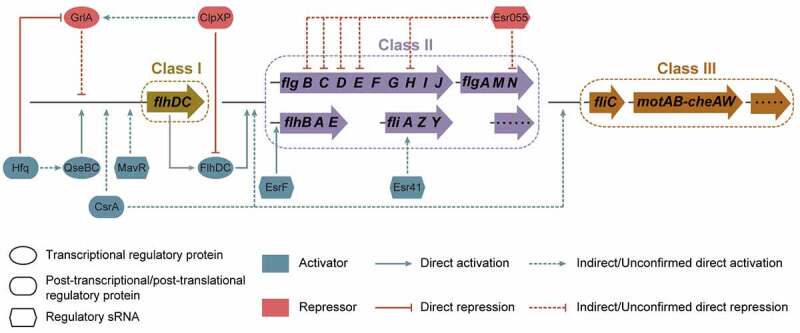
Regulation of flagellar gene expression by post-transcriptional and post-translational regulatory proteins and regulatory small regulatory RNAs (sRNAs) in EHEC O157:H7. Post-transcriptional and post-translational regulatory proteins affecting EHEC O157:H7 motility and flagellar biosynthesis include CsrA, ClpXP, and Hfq. Regulatory sRNAs affecting EHEC O157:H7 flagellar motility and biosynthesis include EsrF, Esr055, Esr41, and MavR.

In addition to transcriptional regulation, post-transcriptional and post-translational regulation play a key role in coordinating flagellar gene expression networks.^72^ Post-transcriptional regulation is highly versatile and adaptable; it controls RNA availability in cellular time and space.^90^ Messenger RNA stability, transport, storage, and translation are largely determined by the interaction of mRNA with post-transcriptional regulatory proteins and sRNAs.^91,92^ Post-translational regulation controls the biochemical alteration of proteins involving generally reversible covalent modification or irreversible processing to regulate their activity, location, or stability.^93,94^ Post-transcriptional and post-translational regulatory proteins known to regulate EHEC O157:H7 motility and flagellar biosynthesis include CsrA, ClpXP, and Hfq (Figure 6 and Table 1).

### CsrA

CsrA is a well-documented RNA-binding protein that post-transcriptionally controls numerous genes and processes.^95,96^ The homodimeric protein CsrA regulates gene expression post-transcriptionally by binding to sites containing the AGGA/ANGGA motif in the leader segment of transcripts to alter their stability and/or translation.^97^ Although the mechanism through which CsrA regulates target gene expression is well documented, the full regulatory impact of CsrA on cellular activities in EHEC O157:H7 is not fully understood. We recently investigated the global effects of CsrA in EHEC O157:H7 using RNA-seq technology, and found that the transcript levels of 53 flagella-related genes were significantly decreased in the *csrA::kan* mutant compared to those in the EHEC O157:H7 wild-type strain.^48^ These downregulated genes comprise both genes required for the synthesis and assembly of flagella as well as transcriptional regulators of flagellar gene expression, such as *flhD, flhC, flgM*, and *fliA*. Subsequent motility assays confirmed that the *csrA::kan* mutant was non-motile on semi-solid agar plates, and the motility defect was rescued by complementation with a low copy number plasmid harboring *csrA*. As expected, the overexpression of *csrA* in EHEC O157:H7 resulted in enhanced bacterial motility. Previous study demonstrated that the CsrA protein of *E. coli* K-12 binds directly to the 5′ segment of *flhDC* mRNA to increase its steady-state level and half-life.^98^ As in *E. coli* K-12, *csrA* disruption in EHEC O157:H7 also results in significant downregulation of *flhDC* gene expression, suggesting that these two strains might share similar motility regulatory mechanisms.^48^

### ClpXP

ClpXP protease is an ATP-dependent bipartite protease responsible for the degradation of some key regulatory proteins (such as RpoS) and aberrant translation products.^99,100^ ClpXP consists of a ClpP protein degradation component and a ClpX component that binds the substrate protein.^101^ In EHEC O157:H7, both FlhD and FlhC proteins accumulated markedly following ClpXP depletion, and their half-lives were significantly longer in the mutant cells, suggesting that ClpXP causes the degradation of FlhD and FlhC proteins, leading to the downregulation of flagellar gene expression.^49^ In addition, ClpXP negatively regulates the transcription of the *flhD* promoter through the GrlR‒GrlA system under conditions in which LEE gene expression is induced. Therefore, the ClpXP protease regulates EHEC O157:H7 motility and flagellar gene expression through two pathways, namely post-translational degradation of the FlhD/FlhC master regulator and transcriptional control of the *flhDC* operon through the LEE-encoded GrlR‒GrlA regulatory system.^49^

### Hfq

The RNA-binding protein Hfq is a global post-transcriptional regulator that is essential for the fitness and virulence of an increasing number of bacterial pathogens.^102^ Hfq promotes interactions between an sRNA and its target mRNA to regulate gene expression; however, Hfq can also function independently by influencing polyadenylation or translation of mRNAs.^103^ Transcriptome and qRT-PCR analyses showed that the deletion of *hfq* in EHEC O157:H7 led to decreased expression of the two-component system *qseBC*,^50^ which is involved in the transcriptional activation of flagellar genes.^43,44^ In contrast, Hfq post-transcriptionally represses the expression of *grlA* that encodes a negative regulator of flagellar gene transcription.^51^ *In vitro* analyses revealed that the Hfq distal face directly binds near the translational initiation site of the *grlA* mRNA and inhibits its translation in an sRNA-independent manner in EHEC O157:H7.^52^ It is therefore conceivable that Hfq can indirectly activate EHEC O157:H7 motility and flagellar gene expression through post-transcriptional regulation of *qseBC* and *grlA*.

## sRNA affecting EHEC O157:H7 motility and flagellar biosynthesis

Bacterial small regulatory RNAs (sRNAs) are generally untranslated RNA sequences ranging from 50 to 300 nucleotides in length.^104^ The majority of sRNAs exert their regulatory effects by binding to target mRNAs, which in turn affect the transcriptional elongation, stability, or translation of mRNAs.^104^ sRNAs have been demonstrated to co-regulate numerous biological processes, including motility, quorum sensing, biofilm formation, stress response, and virulence.^105^ To date, four sRNAs, including EsrF, Esr055, Esr41, and MavR, have been identified to be involved in the regulation of EHEC O157:H7 motility and flagellar biosynthesis (Figure 6 and Table 1).

### EsrF

In our recent work, the sRNA EsrF was identified in a screen for differentially expressed genes/sRNAs from EHEC O157:H7 attached to HeLa cells compared with EHEC O157:H7 grown in DMEM.^106^ EsrF is 85 nucleotides in length and located in an intergenic region between *ntrB* and *glnA*.^53^ *esrF* promoted the expression of several flagellar genes including *flhB, flhD, flhC, fliA*, and *fliC*, which resulted in enhanced EHEC O157:H7 motility.^53^ Among these EsrF-regulated flagellar genes, *flhB* was identified as the direct target of EsrF, which directly binds to the 5′-untranslated region (UTR) of the *flhB* mRNA, which may release the Rho-dependent termination of the *flhB* 5′-UTR, resulting in increased *flhB* expression. A 9-nucleotide crucial motif (5′-GGGAUUUAG-3′) is vital to the ability of EsrF to bind to *flhB* mRNA. Further EMSAs and DNase I footprinting analyses revealed that the nitrogen regulatory protein NtrC directly bound to an 18-base pair motif (5′-CACGGATGAAGCGATCGA-3′; located at −106 to −89 from the *esrF* translational start site) within the *esrF* promoter region to repress the expression of *esrF*.^53^ Furthermore, qRT-PCR analysis revealed that the transcription level of *flhB* was significantly increased in ∆*ntrC* mutant compared to that in the wild-type and complemented strains. Collectively, NtrC senses high ammonium concentrations in the colon to release the inhibitory effect on *esrF*, thereby enhancing its expression and subsequently promoting EHEC O157:H7 motility and colonization.^53^

### Esr055

Similar to the process of discovering *esrF*, we identified the sRNA Esr055 in genomic island OI-93 by re-analyzing our previous transcriptomic data for EHEC O157:H7 during the infection of HeLa cells.^106^ Esr055 is located in an intergenic region between *z3342* and *stx1B*, which encode a 9-O-acetyl-N-acetylneuraminic acid deacetylase and the B subunit of Stx 1, respectively.^54^ The expression of Esr055 is directly activated by the regulator, DeoR, and its expression is positively affected by DNA, which is significantly more abundant in the ileum than in the colon.^54^ Transcriptome analysis revealed that the expression of six flagellar genes, including *flgB, flgC, flgD, flgE, flgH*, and *flgN*, were significantly upregulated in the Δ*esr055* mutant compared with that in the wild-type EHEC O157:H7.^54^ The regulation of these flagellar genes by Esr055 indicated by the transcriptome data was further confirmed by a qRT-PCR analysis. However, the mechanism of how these flagellar genes are regulated by Esr055 remains unclear.

### Esr41

Esr41 (EHEC O157 small RNA #41) is a 70-nt-long sRNA, with a 3′ GC-rich palindrome sequence followed by a long poly(U),^55^ which is essential for functional Hfq-binding.^107^ EHEC O157:H7 harboring a high-copy-number plasmid carrying the *esr41* gene showed increased bacterial motility on agar plates and *fliC* expression at both transcriptional and translational levels.^55^ The enhancement of Esr41-induced bacterial motility was also observed in *E. coli* K-12, indicating that target genes controlled by Esr41 are conserved and present in both EHEC O157:H7 and *E. coli* K-12. Further studies found that Esr41 activated the expression of Class III flagellar genes by indirectly inducing the transcription of *fliA* and thereby enhanced EHEC O157:H7 motility.^56^ Although direct targets of Esr41 remain unknown, Esr41 seems to upregulate *fliA* transcription via a transcriptional regulator whose encoding gene is targeted by Esr41.^56^ FlhDC is the major transcriptional activator responsible for *fliA* expression, and a possible base-pairing region is located between Esr41 and the 5′-UTR of the *flhDC* mRNA.^55^ However, Esr41 does not target *flhD* because the deletion of the possible base-pairing region had no effect on *flhD* expression.^55^ The direct targets of Esr41 involved in regulating EHEC O157:H7 motility and flagellar biosynthesis remain unidentified and need further investigation.

### MavR

MavR (Metabolic and virulence regulatory factor) is a ∼370-bp Hfq-dependent sRNA located within the genomic island OI-48 in the reverse orientation of genes encoding tellurite resistance (*ter*).^57,73^ Bioinformatics analyses revealed that MavR is conserved in diarrheagenic *E. coli* as well as in other pathogenic *Enterobacteriaceae* but is absent in nonpathogenic bacteria.^57^ RNA-seq analyses revealed that the expression of nearly every gene that encodes flagellar biosynthesis or chemotaxis was decreased in the Δ*mavR* mutant compared to wild-type EHEC O157:H7 during aerobic growth.^57^ The RNA-seq results were further confirmed by a qRT-PCR analysis of four differentially expressed flagellar genes, including *flhD, fliK, fliC*, and *cheA*. Further analyses found that MavR promotes flagellar/chemotaxis gene expression by influencing FlhD expression post-transcriptionally. Consistent with the gene expression results, the motility of the Δ*mavR* mutant on semi-solid LB agar was slightly decreased compared to that of wild-type EHEC O157:H7, and this difference could be rescued upon complementation.^57^

## Conclusions and future perspectives

Flagellum-dependent motility plays an essential role in EHEC O157:H7 colonization and pathogenesis in the host intestinal tract.^108,109^ However, flagellar protein synthesis comes at a cost to the bacterium in terms of growth rate and fitness within the host.^110^ Furthermore, flagellin is a potent antigen that induces an immune response by eliciting the secretion of proinflammatory chemokines in human intestinal epithelial cells.^111^ Therefore, flagellar gene expression must be tightly regulated to occur at the appropriate time and place during different stages of infection.^72^ EHEC O157:H7 bacteria have evolved diverse regulatory mechanisms to control the expression of flagellar genes in response to specific intestinal environmental cues, including transcriptional regulatory proteins, post-transcriptional and post-translational regulatory proteins, and regulatory sRNAs. During early infection, EHEC O157:H7 upregulates flagellar genes in response to intestinal environmental signals, ultimately increasing bacterial flagellar biosynthesis and motility.^10^ Subsequently, the increased motility enables the pathogen to reach and adhere to colonization sites in the host intestine. After successful infection, flagellum-dependent motility becomes less critical, and EHEC O157:H7 then downregulates flagellar gene expression to inhibit flagellar biosynthesis, not only to save energy but also to minimize host immunity.^10^ This finely tuned strategy allows EHEC O157:H7 to precisely control the timing of activation of flagellar genes at the intended site of action, maximizing its virulence and pathogenicity. Nevertheless, only a few intestinal environmental signals affecting flagellar gene expression have been identified thus far, and the precise regulatory mechanisms remain largely unknown. Thus, whether other intestinal environmental cues in the human intestine elicit flagellar biosynthesis remains to be established in future studies.

A comparative genomic analysis showed that EHEC O157:H7 genome contained 177 genomic islands, termed O islands (OIs) that are not present in the genome of nonpathogenic *E. coli* K-12.^73^ These islands comprise the main known virulence elements of EHEC O157:H7, including LEE pathogenicity island (OI-148) and Shiga toxin-converting phages (OI-45 and OI-93).^2,112^ The functions of several other islands have also been established in recent years, revealing more regulatory proteins and sRNAs associated with EHEC O157:H7 motility and flagellar biosynthesis. The *gmrA* gene on OI-29 and *z5898* on OI-172 were found to encode the transcriptional activators of flagellar biosynthesis and bacterial motility, while *fmrA* on OI-1 was found to encode a repressor.^29,31,35^ In addition, of the four known sRNAs that regulate EHEC O157:H7 motility and flagellar biosynthesis, two are located on OIs, with Esr055 on OI-93 and MavR on OI-48.^54,57^ Nevertheless, the majority of OI genes have not been assigned a function, and their effect on EHEC O157 motility and flagellar biosynthesis is still largely unknown. Moreover, the majority of current studies have mainly focused on investigating the relationship between core genome-encoded regulatory factors and the flagellar biosynthesis of EHEC O157:H7. In contrast, the studies on the functions of OI-encoded regulatory factors associated with flagellar biosynthesis have been relatively limited. Therefore, future studies should focus on the effect of OI-associated genes on bacterial motility and flagellar biosynthesis, which might facilitate an improved understanding of the mechanisms of EHEC O157:H7 pathogenicity and adaptation to different environmental conditions.

Diarrheal diseases caused by EHEC O157:H7 are an important public health problem worldwide. Outbreak surveillance data from the Center for Disease Control and Prevention (CDC) reported that EHEC O157:H7 causes more than 73 000 illnesses, 2100 hospitalizations, and 60 deaths annually in the United States.^113^ The annual cost of illness due to *E. coli* O157:H7 infections, including lost productivity, medical care, and premature deaths, was 405 million dollars.^113^ Intestinal tract infections caused by EHEC O157:H7 cannot be treated effectively by current antibiotic therapies because the antibiotics are either ineffective, cause severe dysbiosis of the intestinal microbiota, or trigger serious complications, such as HUS from antibiotic-induced Stxs release.^114,115^ Thus, new strategies must be employed to develop safe and effective antimicrobial therapies for the treatment of EHEC O157:H7 infections. Considering that flagellum-mediated motility plays diverse roles in EHEC O157:H7 pathogenesis and the deletion of many genes encoding regulatory proteins and sRNAs would significantly decrease EHEC O157:H7 motility and flagellar biosynthesis, these genes may hence be used as potential targets for the development of novel therapeutics for the treatment of EHEC O157:H7 infections.
